# A dataset for spectral radiative properties of black poly(methyl methacrylate)

**DOI:** 10.1016/j.dib.2022.108097

**Published:** 2022-03-26

**Authors:** Farid Alinejad, Hadi Bordbar, Michalina Makowska, Simo Hostikka

**Affiliations:** School of Engineering, Aalto University, Finland

**Keywords:** Black PMMA, Optical constants, Spectral transmissivity, Absorption coefficient, ATR-FTIR spectroscopy, Spectral modeling of thermal radiation

## Abstract

The main objective of this article is to provide the spectroscopic radiative properties of black poly(methyl methacrylate) (PMMA) including transmissivity, absorption coefficient, and complex index of refraction. To perform the required spectroscopy, four ultra-thin samples with thicknesses of 33±1.3, 50±1.3, 65±1.0, and 73±1.5 µm were prepared. Then, by using UV–Vis-NIR and FTIR spectrometers, the spectrum of transmissivity was measured for the wavelength regions of 0.25 to 2.5 and 2.5 to 25 µm, respectively. Applying modified Beer’s law, the absorption coefficient of black PMMA was extracted. To obtain the refractive index, first the reflectivity of the 6 mm sample of black PMMA measured by UV–Vis-NIR spectrometer. Then, applying the Kramers–Kronig transform and Fresnel relation, the refractive index of black PMMA was extracted. To investigate the effect of temperature on the absorbance of the material, ATR-FTIR spectroscopy was done for the temperatures below melting point (i.e., 160 ${}^{{}^{\circ }}$C). Finally, a set of data for effective absorption coefficient as a function of depth from the sample surface and source temperature was proposed to be used in pyrolysis modeling.

## Specifications Table

**Table:** 

Subject	Engineering
Specific subject area	Pyrolysis modeling in fire simulations
Type of data	Table
How the data were acquired	Transmissivity, reflectivity, and absorbance data were measured by Cary 5000 UV–Vis-NIR, Nicolet iS50 FTIR, and Nicolet iS50 FTIR-GladiATR spectrometers, respectively. Then, absorption coefficient was extracted using measured transmissivity and applying modified Beer’s law and linear regression. To extract the refractive index, Kramers-Kronig transform and Fresnel relation were applied. Finally, effective absorption coefficient was calculated by applying the Beer’s law to the black-body irradiation weighted total transmissivity.
Data format	Raw, Analyzed
Description of data collection	Transmissivity data were measured for the sample thicknesses of 33±1.3, 50±1.3, 65±1.0, and 73±1.5 µm using spectrometers. Absorbance at different temperatures were measured applying the sample thickness of 175 µm using FTIR-ATR spectrometer. Using a sample thickness of 6 mm, the total and diffuse reflectivities were measured using UV–Vis-NIR spectrometer.
Data source location	Aalto university, Espoo, Finland.
Data accessibility	Repository name: Spectral radiative properties of black PMMA
	Data identification number: 10.17632/8wpgzrp33s.1
	Direct URL to data: https://data.mendeley.com/datasets/8wpgzrp33s/1
Related research article	F. Alinejad, H. Bordbar, M. Makowska, S. Hostikka, Spectroscopic determination of the optical constants and radiative properties of black PMMA for pyrolysis modeling, International Journal of Thermal Sciences 176 (2022) 107501. https://doi.org/10.1016/j.ijthermalsci.2022.107501

## Value of the Data

• Black PMMA is one of the mostly used material in the fire research and its spectral radiative properties for the entire UV-Vis-NIR and FTIR regions has not published yet.
• These datasets can be implemented in pyrolysis models for more realistic fire simulations. The other applications of these data can be in the polymer research.
• The datasets can be applied in spectral or gray modeling of thermal radiation in pyrolysis modeling.

## 1 Data Description

The excel file “Transmissivity_50µm_UV–Vis-NIR.xlsx” presents the measured transmissivity of 50 µm black PMMA sample for the UV–Vis-NIR region (i.e., the wavelength region of 0.25 to 2.5 µm). The data file of “Transmissivity_73µm_UV–Vis-NIR.xlsx” gives the measured transmissivity of 73 µm black PMMA sample for the UV–Vis-NIR region.

The measured transmissivity of black PMMA for the thickness of 33 µm for the wavelength region of 2.5 to 25 µm is given by “Transmissivity_33µm_FTIR.xlsx” file. For the thickness of 50 µm, the measured transmissivity in the wavelength region of 2.5 to 25 µm is presented in “Transmissivity_50µm_FTIR.xlsx”. Finally, the data file of “Transmissivity_65µm_FTIR.xlsx” gives the measured transmissivity of 65 µm black PMMA sample in the wavelength region of 2.5 to 25 µm.

The data files of “Total reflectivity.xlsx” and “Diffuse reflectivity.xlsx” present the measured total and diffuse reflectivities in the wavelength region of 0.5 to 2 µm, respectively.

The measured absorbance spectra of black PMMA in the wavelength region of 2.5 to 25 µm for the temperature of 50 ${}^{{}^{\circ }}$C is given in “Absorbance_50C_FTIR-ATR.xlsx”. For the temperature of 150 ${}^{{}^{\circ }}$C, the spectra of absorption at same wavelength region is presented in “Absorbance_150C_FTIR-ATR.xlsx”.

The calculated absorption coefficient of black PMMA from the measured transmissivities for the wavelength range of 0.25 to 25 µm is given by “Absorption coefficient.xlsx”.

The absorptive index spectrum of black PMMA for the wavelength region of 0.25 to 25 µm is presented in “Absorptive index.xlsx”. The data file of “Refractive index.xlsx” gives the refractive index of black PMMA for the wavelength region of 0.25 to 25 µm.

The data file “Effective absorption coefficient.xlsx” in the repository gives the effective absorption coefficient for depth interval of 0.1 to 1000 mm and source temperature interval of 500 to 2000 K.

## 2 Experimental Design, Materials and Methods

### 2.1 Sample preparation and measurement devices

**Fig. 1:**
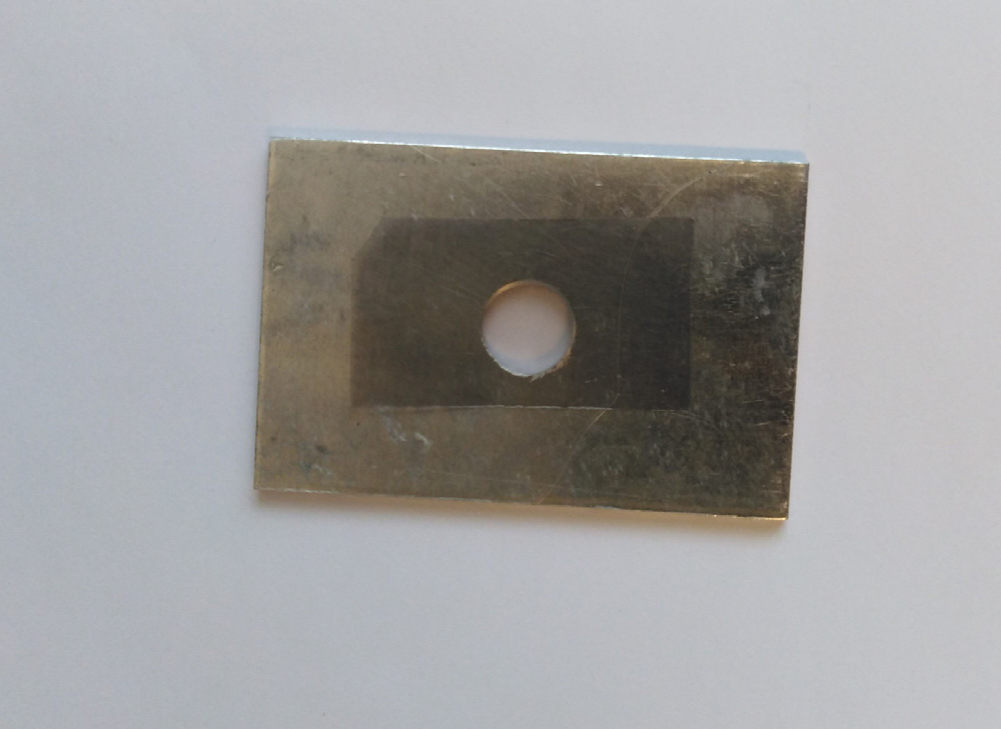
Applied sample holder with one of the samples in spectroscopy.

The black PMMA manufactured by Evonic with tradename of ACRYLITE® cast black 9H01 GT was used in the measurements. The thickness of the original sample was 6 mm. Primary measurements of the transmissivity with FTIR spectrometers showed that due to the strong absorption of black PMMA, very thin samples of black PMMA should be used in spectroscopy. To determine the needed thicknesses of the material, several measurements of transmissivity in FTIR range were done using samples with thicknesses between 100 and 200 µm. Considerable part of the transmissivity spectrum could not be captured using these thicknesses. The results of these measurements showed that the needed samples should be as thin as possible. Due to the manufacturing limitations and the brittleness of black PMMA, 33 µm was the lowest attainable thickness. Therefore, using the grinding method, four different samples with thicknesses of 33±1.3, 50±1.3, 65±1.0, and 73±1.5 µm were produced. To eliminate the surface roughness effect in measurements, a polishing was done for all the samples. The main problem with polishing of very thin samples, was in fixing them on the sample holder. To properly fix the ultrathin samples to the holders for polishing, a two-sided tape was used, and after the polishing, liquid ethanol was used to dissolve the tape for detaching the samples from sample holder. To report the uncertainty for the thickness of the samples, at least the thickness of 10 different points of each sample was measured. To insert the samples into spectrometers, some sample holders were made as shown in Fig. 1.

To measure the optical constants and radiative properties of black PMMA for a wide enough wavelength region, measurements were done using UV–Vis-NIR and FTIR spectrometers. The measurement of the transmissivity for the wavelength range of 0.25 to 2.5 µm was done for the 50 and 73 µm samples of black PMMA using the Cary 5000 UV–Vis-NIR spectrometer from Agilent Technologies [1] with resolution of 1 nm. Upper bound of wavelength (i.e., 2.5 µm) for UV–Vis-NIR measurements was selected to skip the noisy data for higher wavelengths. To measure the transmissivity for the wavelength range of 2.5 to 25 µm for the sample thicknesses of 33, 50, and 65 µm, the Nicolet™ iS50 FTIR spectrometer [2] was applied with a resolution better than 0.09 cm${}^{-1}$. To measure the total and diffuse reflectivity of black PMMA in UV–Vis-NIR region, the original sample with thickness of 6 mm was used. The reflectivities were measured using the Cary 5000 UV–Vis-NIR spectrometer from Agilent Technologies [1] with resolution of 1 nm. Finally, for the Attenuated Total Reflectance (ATR) measurement, samples with thickness of 175 µm were prepared. Measurements were done using Nicolet™ iS50 FTIR spectrometer [2] with the accessory GladiATR. To set the sample temperature, a heating stage manufactured by Pyke technologies was applied. Using this device we can increase the sample temperature up to 200 ${}^{\circ }$C. Measurements were done up to sample temperature of 150 ${}^{\circ }$C to avoid the melting of the samples.

### 2.2 Methods

After measuring transmissivity for different samples, Eq.  (1) is applied for the calculation of absorption coefficient [3]:

(1) $$\tau_{\lambda }={\left(1-\rho \right)}^{2}exp\left(-\kappa_{\lambda }L\right)\Rightarrow \ln\left(\tau_{\lambda }\right)=-\kappa_{\lambda }L-2\ln\left(1+\rho \right)$$

where $\tau_{\lambda }$, $\kappa_{\lambda }$, L, λ, and ρ are transmissivity, absorption coefficient, sample thickness, wavelength, and reflectivity at the sample surface, respectively. To derive the Eq. 1, internal reflections of the radiation was considered with assuming constant reflectivity at interfaces. Determining the slope of $\ln(\tau_{\lambda })$ plot with respect to L using linear regression gives the absorption coefficient. Due to opaqueness of the sample for the wavelength regions of 5.72 to 5.85 µm and 8.3 to 8.85 µm, transmissivity of black PMMA could not be captured applying the current sample thicknesses. For these ranges, values are estimated using extrapolation by curve fitting. With having spectrum of absorption coefficient, absorptive index ($k_{\lambda }$) of black PMMA is determined using:

(2) $$k_{\lambda }=\frac{\kappa_{\lambda }\lambda }{4\pi }$$

To determine the refractive index of the material, we used the fact that absorptive and refractive indexes of the materials are related to each other through Kramers-Kronig transform [3]:

(3) $$n_{v}=n_{\infty }+\frac{2}{\pi }P\int_{0}^{\infty }\frac{v^{\prime }k_{v^{\prime }}}{v^{\prime 2}-v^{2}}dv^{\prime }$$

(4) $$k_{v}=-\frac{2v}{\pi }P\int_{0}^{\infty }\frac{n_{v^{\prime }}-n_{\infty }}{v^{\prime 2}-v^{2}}dv^{\prime }$$

Having the absorptive index, the modified Beer’s law (i.e., Eq.  (3)) gives the refractive index. There are two issues in using of Eq.  (3): 1) lack of experimental data for very low and very high wavenumbers and 2) lack of information about $n_{\infty }$. The first issue could be solved with the fact that the effect of higher wavenumber on the values of refractive index is negligible and extrapolation can be applied to the short wavenumbers [4]. To obtain $n_{\infty }$, with having reflectivity, refractive index can be calculated using Fresnel’s relation [3]:

(5) $$\rho_{s\lambda }=\frac{{\left(n_{\lambda }-1\right)}^{2}+k_{\lambda }^{2}}{{\left(n_{\lambda }+1\right)}^{2}+k_{\lambda }^{2}}$$

By minimizing the calculated refractive index using Eqs.  (3) and (5), the value of $n_{\infty }$ is determined.

For gray modeling of thermal radiation, an effective absorption coefficient ($\kappa_{\mathrm{eff}}$) was calculated as a function of depth from the sample surface and source temperature using:

(6) $$\tau_{\mathrm{tot}}=\frac{1}{I_{b}\left(T_{s}\right)}\int_{0}^{\infty }I_{b\lambda }\left(T_{s}\right)\exp\left(-\kappa_{\lambda }x\right)d\lambda =\exp\left(-\kappa_{\mathrm{eff}}\left(x,T_{s}\right)x\right)$$

(7) $$\kappa_{\mathrm{eff}}\left(x,T_{s}\right)=-\frac{1}{x}\ln\left(\tau_{\mathrm{tot}}\right)$$

where $\tau_{\mathrm{tot}}$, x, $I_{b\lambda }$, and $I_{b}$ are total transmissivity, depth from the sample surface, spectral blackbody radiation intensity, and total blackbody radiation intensity, respectively.

## CRediT authorship contribution statement

**Farid Alinejad:** Conceptualization, Methodology, Software, Resources, Formal analysis, Investigation, Data curation, Writing – original draft, Visualization. **Hadi Bordbar:** Conceptualization, Methodology, Resources, Writing – review & editing, Investigation, Supervision, Project administration. **Michalina Makowska:** Conceptualization, Methodology, Resources, Formal analysis, Data curation, Writing – review & editing, Investigation. **Simo Hostikka:** Conceptualization, Methodology, Writing – review & editing, Supervision, Project administration, Funding acquisition.

## Declaration of Competing Interest

The authors declare that they have no known competing financial interests or personal relationships that could have appeared to influence the work reported in this paper.

## Data Availability

Spectral radiative properties of black PMMA (Original data) (Mendeley Data).
